# TOR-dependent regulation of the yeast homolog of the juvenile Batten Disease-associated gene *CLN3*

**DOI:** 10.15698/mic2026.03.872

**Published:** 2026-03-11

**Authors:** Vijaykumar Pillalamarri, Samuel W.M. Gatesy, Amanda E. Grassel, Lucienna Wolf, Justin P. Whalley, David M. Mueller

**Affiliations:** 1 Discipline of Biochemistry and Molecular Biology, Center for Genetic Diseases, The Chicago Medical School, Rosalind Franklin University of Medicine and Science, 3333 Green Bay Road, North Chicago, IL, 60064, United States of America; 2 Discipline of Microbiology and Immunology, Center for Cancer Cell Biology, Immunology, and Infection, The Chicago Medical School, Rosalind Franklin University of Medicine and Science, 3333 Green Bay Road, North Chicago, IL, 60064, United States of America

**Keywords:** BTN1, *CLN3*, Batten Disease, translational regulation, amino acid starvation

## Abstract

The Juvenile form of Batten disease is a neurodegenerative disease with symptoms starting in the first decade and ending in death in the third decade of life. The gene defective in this form of Batten disease, *CLN3*, is conserved in eukaryotes, suggesting that the gene product serves a basic function in the cell, though the function is unknown. We have investigated the expression and regulation of the yeast homolog BTN1. Reanalysis of publicly available gene expression data suggests that transcription of BTN1 increases in response to oxidative stress, treatment with rapamycin or arsenate, amino acid starvation, and sporulation conditions. Similar to GCN4, there are upstream open reading frames (uORF) in front of BTN1, suggesting translational regulation. We developed reporter strains in which the HIS3 open reading frame replaced that of the BTN1 gene, with and without the uORFs. These reporters show that one or more of the uORFs decrease the expression of the HIS3 reporter. When expressed in the reporter strain using a high copy vector, GCN3, tRNA ${}^{\mathrm{Arg}}$ , and tRNA ${}^{\mathrm{Leu}}$ , increase expression, suggesting the involvement of the TORC1 pathway. BIT61 abuts BTN1 but is encoded on the opposite strand; 3’ RACE analysis indicates that the mRNA of BIT61 overlaps with that of BTN1. BIT61 is involved in the TORC2 pathway, which interacts with the TORC1 pathway, suggesting a possible cis-acting mechanism of co-regulation. Lastly, we demonstrate that a yeast strain with a null mutation in BTN1 is sensitive to selective amino acid starvation, further supporting the association of BTN1 with TORC1.

## INTRODUCTION

Neuronal ceroid lipofuscinoses (NCL, commonly known as Batten disease) are a group of progressive neurodegenerative, lysosomal storage disorders inherited in an autosomal recessive manner 1. Accumulation of auto-fluorescent, lipofuscin-like ceroid material in lysosomes of neuronal as well as non-neuronal cells, an aging-related characteristic, is detected in most of the NCLs 2, 3. Symptoms start to appear in various stages of life depending on the NCL, starting with vision loss, cognitive and motor function impairment, and ultimately leading to premature death 1. The primary treatments available for NCLs are aimed at alleviating symptoms, with a few exceptions; for example, enzyme replacement therapy, which only slows the progression of CLN2 disease 4.

NCLs are the result of defective forms of Ceroid Lipofuscinosis Neuronal protein 5, 6. Juvenile Neuronal Ceroid Lipofuscinosis (JNCL), caused by a spectrum of mutations in *CLN3*, either alone or together, is one of the most prevalent of the 13 forms of NCLs 1, 7–10. In JNCL patients, the symptoms start as early as age 5 with vision loss, with progressing symptoms, ultimately leading to death in their twenties or thirties 7. Vision loss is common in neurodegenerative diseases, including Parkinson’s and Alzheimer’s 11. A common characteristic in JNCL is the presence of fingerprint-like storage material in the lysosome. The majority of the protein in the storage material consists of subunit-c of ATP synthase 12, 13, while the majority of the lipid component is cholesterol 14. Subunit c of the ATP synthase is encoded in the nucleus, and the pre-protein contains a mitochondrial targeting sequence that is removed on import into the mitochondrion. Subunit-c in the storage material is in the mature form, indicating that its presence is the result of mitophagy.

CLN3 is highly conserved across eukaryotes, from yeast (BTN1, also known as YHC3) to humans, indicating an important role in fundamental cellular functions. Among the 13 types of NCLs, six affected disease genes have a yeast homolog, suggesting conservation of a core pathway. Expressing human CLN3 in a btn1 Δ yeast strain restores Btn1p function 15, 16. CLN3 and Btn1p are localized to lysosomes and the yeast homolog vacuole 17–20, although CLN3 has also been reported to be found in the plasma membrane, Golgi apparatus, endosomes, nucleus, and mitochondria 21–25. It is proposed to be involved in various physiological processes, including maintaining vacuolar pH 15, 26, 27, amino acid transport 28, chaperone activity 29, apoptosis 30, autophagy 31, and more. Recently, CLN3 has been shown to be essential for removing glycerophosphodiesters from the lysosome, and patients with JNCL have elevated levels of glycerophosphoinositol in their cerebrospinal fluid 32. However, even after three decades since the discovery of CLN3, its function remains unknown, making *CLN3* an “orphan” gene.

GCN4 is a bZIP transcriptional activator that controls amino acid biosynthesis via a process called General Amino Acid Control 33. GCN4 is positively regulated by GCN1, GCN2, and GCN3 genes, and negatively regulated by several general control derepressible genes (GCD) 33, 34. Yeast GCN4 was the first gene identified to contain uORFs 35, 36. GCN4 contains four uORFs through which GCN and GCD proteins regulate GCN4 expression 33. Under normal conditions, the uORFs block translation 33, 35, 37. During amino acid starvation, Gcn2p phosphorylates eIF2 α , preventing its recycling, which activates GCN4 34. When the uORFs are removed or mutated into non-AUG codons, GCN4 is derepressed and becomes constitutively active 33.

Gcn2p has a sequence similar to a histidyl-tRNA synthetase, which is thought to bind uncharged tRNA ${}^{\mathrm{His}}$ in response to histidine starvation 38. However, genome-wide analysis of tRNA charging and activation shows that more than just uncharged tRNA ${}^{\mathrm{His}}$ can activate Gcn2p 39.

Rapamycin was initially discovered as an antifungal agent and was later found to have immunosuppressive and growth-inhibitory effects in mammalian cells. The target of rapamycin, TOR, was first identified in yeast as TOR1 and TOR2 40, 41. Homologs are present in mammalian cells 42, 43 and are named mTOR, for mechanistic TOR. mTOR is a serine/threonine protein kinase that forms two major complexes with different functions, called mTORC1 and mTORC2 42. TORC1 is a key regulator in nutrient signaling, including amino acids and glucose, and controls cell growth. TORC1 is recruited to the yeast vacuole (lysosome) by the EGO (escape from growth arrest) complex, where it manages TORC1 activity 42, 44, 45. The functional homolog of the yeast EGO complex is believed to be the Ragulator complex 42. SLC38A9 is a member of the Ragulator complex and acts as an arginine sensor necessary for the activation of TORC1 46.

Like GCN4, BTN1 has uORFs, five within the first 180 base pairs upstream of the ORF, suggesting translational regulation of the gene. In this study, we demonstrate with the HIS3 reporter that one or more of the uORFs decrease expression, indicating that BTN1 is controlled at the translational level, likely during amino acid starvation conditions. Consistent with this, overexpressing Gcn3p, tRNA ${}^{\mathrm{Arg}}$ , and tRNA ${}^{\mathrm{Leu}}$ induces HIS3 reporter expression, suggesting a potential role in the TORC1 pathway. Additionally, 5’ RACE results reveal multiple transcription start sites for both BTN1 and BIT61, pointing to another layer of complex transcriptional regulation.

## RESULTS

### Analysis of BTN1 expression

The yeast community has studied the transcriptional landscape of the yeast genome after exposure to various conditions. We examined 603 datasets listed in the Saccharomyces Genome Database (SGD) 47, to explore how BTN1 is regulated at the transcriptional level to help determine Btn1p’s role in the cell. After filtering the datasets that did not have genome-wide expression or had anomalous gene expression levels (see Materials and Methods). We selected and analyzed the top 15 datasets in which BTN1 expression varied the most (**Table 1**). The analysis focused on genes whose expression levels changed—either increased or decreased—under different conditions compared to the control. Most datasets where BTN1 expression was most varied were related to conditions subjecting yeast to stress responses, including oxidative and osmotic stress, amino acid starvation, rapamycin treatment, carbon starvation, and sporulation. For each dataset, we clustered the genes into 16 groups based on expression pattern using the K-means algorithm. We performed pathway enrichment (Gene Ontology, GO, Biological Processes) analysis on gene clusters containing BTN1 and common genes that are present in at least four of the 15 datasets, suggesting an involvement of Btn1p in these processes. The top hits were DNA repair and DNA metabolic process (Figure S1). Other significant biological processes included cellular protein modification and protein catabolic processes.

We also performed pathway enrichment (GO Biological Processes) for each of the 15 datasets on the genes in the cluster where BTN1 is present. The processes discussed above (Figure S1) are found in 20 most enriched GO terms across all 15 datasets (Table S1). Other relevant pathways include the TOR pathway (GO:0031929), which is enriched in eleven datasets, and the general amino acid response (GO:0034198), enriched in twelve datasets (Table S1). Overall, this data, as seen in Table 1, suggests that Btn1p is regulated under stress conditions, including amino acid starvation and activation of the TORC1 pathway.

**Table 1:** List of the conditions under which the expression of BTN1 is most variable. BTN1 levels are increased in stress conditions. Note: There were a total of 15 datasets (n) and 19 conditions or time points assessed. The datasets were all microarray data. When replicates were present, the expression values were averaged. NA: Not available. We have cited the paper from which each dataset originates and, where available, the GEO accession number.

**Conditions**	**n**	**GEO accession**	**Fold change in BTN1 expression**
Amino acid starvation 48	1	NA	1.9

Rapamycin treatment 48	NA	3.3 (30 min) 1.6 (60 min)

Heat shock 49, 50	2	GSE40073	2.3 (WT) 3.1 (disomy X)

GSE38478		3.0

Hydrogen peroxide 50	GSE38478	6.1

Arsenic treatment 51, 52	2	NA	3.3

GSE6068	2.3

Sporulation media 53–56	5	GSE3814	2.3

		GSE3820	2.0

		NA	2.8

GSE24675	2.8

GSE7393	2.2

0.4M NaCl in Glucose limited chemostat medium 57	1	GSE6302	2.0

Calcofluor white 58	1	GSE4049	4.2

Histone H4 depletion 59	1	NA	2.8

Spt6 mutant 60	1	GSE12272	2.2

snf2 Δ 61	1	NA	1.5

### Organization of the BTN1 and BIT61 genes and their transcripts

BTN1 is located on chromosome 10, while BIT61 is on the opposite strand, separated by only 51 bases between their open reading frames. Because BIT61 is a subunit of TORC2 62, we examined the regulation of both genes.

**Figure 1:**
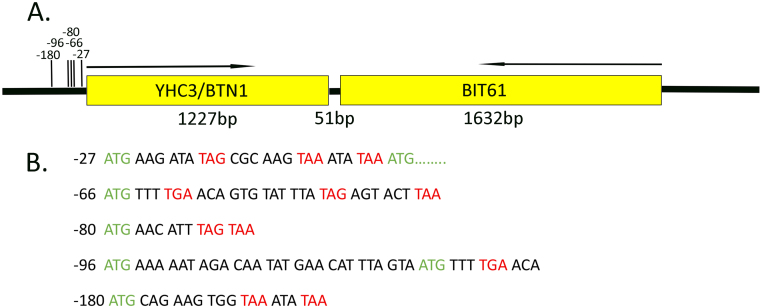
Genomic organization of the BTN1 and BIT61 Locus. **(A)** The organization of the BTN1and BIT61 loci. The arrows indicated the direction of transcription. **(B)** The sequence of the five uORFs. The uORFs are all in frame with BTN1, except uORF-80.

Figure 1 shows the gene layout at the locus, highlighting the five closest uORFs before the BTN1 ORF. BTN1 has five uORFs within 192 bp of its ORF. The uORFs are located at −27, −66, −80, −96, and −180. All except uORF-80 are in frame with BTN1. uORF-27 contains three in-frame stop codons at −18 (TAG), −9 (TAA), and −3 (TAA). Given the ATG, the chance of finding three stop codons within the following eight codons is about 2%. uORF-66 is in frame with BTN1 but includes six stop codons, with the first at −60 (TGA) and the second at −45 (TAG). uORF-80 is out of frame with BTN1 and has two consecutive stop codons at −71 (TAG) and −68 (TAA). uORF-96 is in frame with uORF-66 and has no stop codons until −60. uORF-180 contains stop codons at −168 and −162 (TAA). Additionally, there are uORFs with BIT61, though their role in gene expression has not yet been studied. The presence of uORFs upstream of BTN1 is similar to that of GCN4, which is regulated by translational control and involved in the general amino acid response, especially during amino acid starvation. To clarify the role of the uORFs, we modified the parent strain by removing the uORFs. This was achieved by changing the “T” in the “ATG” start codon to “A” using CRISPR technology. We used this strain to determine whether the changes affected the level of the mRNA or the transcriptional initiation sites under normal growth conditions and after treatment with rapamycin and amino acid starvation.

**Figure 2:**
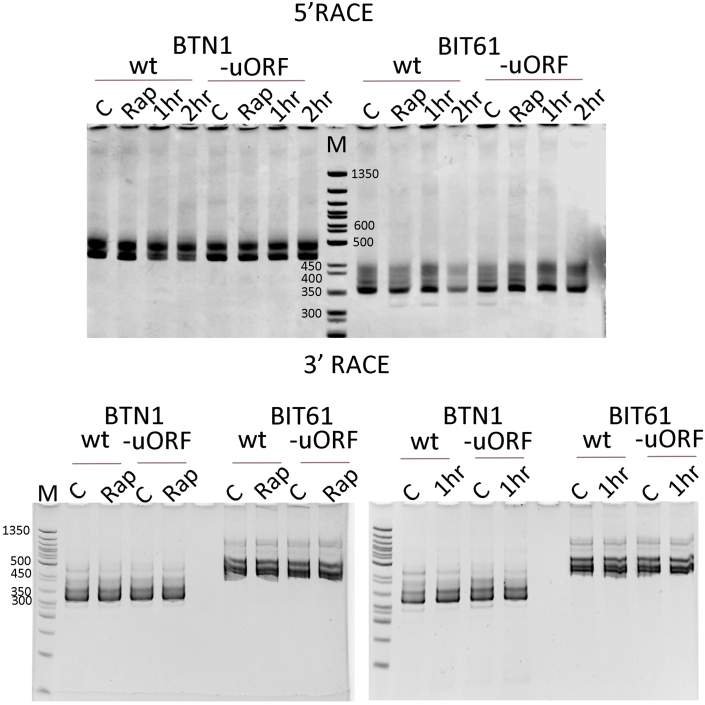
5’ and 3’ RACE products for BTN1 and BIT61. The 5’ and 3’ RACE products were obtained from RNA isolated from cells treated with rapamycin (Rap) or starved for amino acids for 1 or 2 hours. The products were separated on a 4% polyacrylamide gel and stained with ethidium bromide.

First, we performed 5’ and 3’ RACE analysis. Figure 2 shows the products generated after 5’ and 3’ RACE (Rapid *Amplification* of cDNA Ends) using RNA isolated from wild type (DBY746) and minus uORF 1–5 (AAY002), following growth in the presence of rapamycin (Rap) and after amino acid starvation for 1 and 2 hours. In this experiment, we employed the “template switching” method 63, 64 with primers at 312 for BTN1 and 217 for BIT61, producing the largest fragments of approximately 500 bp for BTN1 and 450 bp for BIT61. This size includes the template switching primer (36 bp), positioning the larger fragment at about −152 for BTN1. For BIT61, the 5’ site maps to roughly −197. However, when we sequenced these products, the DNA sequence was clear up to around −17 and then overlapped with multiple reads extending to −80 and beyond. For BIT61, the DNA sequence read to approximately −42 and then showed overlapping reads extending to about −100 and beyond. In both cases, minor reads covered another 100 bases. As demonstrated by the gels, we did not observe any significant differences between the RNA isolated from the strains after growth with rapamycin or after amino acid starvation. The Sanger DNA sequence results for the 3’ RACE products were less complex than those for the 5’ results. For the BTN1 gene, +87C (1227C) and +88G (1228G) were the main sites. This transcript extends 37 bases into the neighboring BIT61 ORF. For the BIT61 gene, +31A (1663A) was the primary site, but transcripts were also seen as far as +68C (1700C). This transcript extends 17 bases into the BTN1 ORF. As a result, the overlap between the BTN1 and BIT61 transcripts ranges from 67 to 103 bases.

Because the Sanger sequencing reactions had overlapping sequences for the 5’ RACE, we performed Next Generation Sequencing on the 5’ RACE products. We also changed the methodology from template switching to a traditional method by adding poly-dA to the 3’ end of the newly synthesized cDNA. The poly-A sequence is then used as a partner for the QT-RACE primer during second-strand synthesis. The QT-RACE primer contains a 35-base sequence in addition to the poly-dT, serving as a hybridization site for the two primers, Qo and Qi. We used poly-A RNA instead of total RNA to enrich for mRNA.

**Figure 3:**
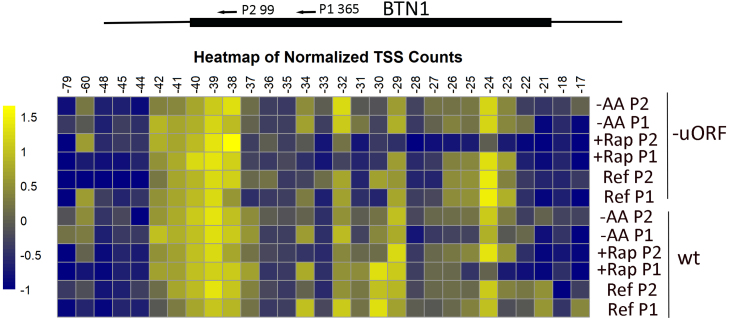
Heatmap displaying the normalized counts of transcription start sites of BTN1. The cells were incubated as noted in Figure 2, with rapamycin added to the media (+Rap), amino acid starvation for 1 hr (-AA), and a reference condition with growth in complete synthetic media. RNA was isolated (see Materials and Methods), and Next Generation Sequencing (NGS) of 5’ RACE products after amplification with BTN1 reverse primers at 99 (P2) or 365 (P1). The scale indicates the log ${}_{10}$ of the normalized count percentage for each fragment.

Figure S2 displays the results of 5’ RACE using the traditional method. The results are more complex because the tailing reaction with dATP adds a variable number of nucleotides. For NGS sequencing, we analyzed only DNA reads that included the sequence of the QT-RACE primer, which also identified the poly (A) tail. Therefore, all reads were primed with poly-dT and amplified using the QT-RACE primer. These results are summarized in Figure 3 as a heat map. The primary transcripts mapped to −24A, −29A, −32C, and −39A, with groupings centered around −24, −32, and −40. The first three uORFs start at −27, −66, and −96, placing uORF-27 and uORF-66 within primary transcripts. About 75% of the mapped transcripts included uORF-27, with a significant portion also including uORF-66. We observed minor transcripts starting at −141 and extending up to −159, indicating that the first four uORFs are present in primary transcripts but at low levels under the tested conditions.

The 5’ RACE results for the BIT61 gene were easier to interpret from Sanger DNA sequence chromatograms. The main, shortest transcript starts at −45G, but we also identified transcripts starting at −49A, −68T, −95G, and −168A.

These results closely match published studies 65, 66. When grown in YPD and YPGal media, the 5’ transcripts started at −55, and −39 for BTN1 and −33 and at −78 for BIT61, respectively 66. In the first study, the 5’ end of BTN1 was mapped to −90, which is outside the main observed sites but still within the observed range 65. For BIT61, the 5’ ends were mapped at −33 for YPD and −78 for YPGal, and the 3’ ends were mapped at ＋30 and ＋58 bases past the ORF, respectively 66. Our cells were all grown in YPD media and harvested at mid-log stage. For amino acid starvation, cells were grown in YPD until mid-log phase and then shifted to minimal media lacking amino acids.

Next, we measured the relative transcript levels of BTN1 and BIT61 loci during the mid-log phase, with rapamycin added for one hour in YPD media and after one hour of amino acid starvation in minimal media. We analyzed the wild-type strain and AAY002, which lacks the five uORFs. Expression was normalized to two common genes, ACT1 and PDA1. Table 2 shows the results of this analysis. Overall, there was no significant change after adding rapamycin, although BTN1 expression appears slightly higher in AAY002 compared to the parent strain. Rapamycin had a small effect on the expression of AAY002, with a similarly small impact on the parent strain. The RNA data after amino acid starvation were more variable across the four experiments (n = 4), but this suggests that amino acid starvation increased BTN1 levels in the wild-type strain and, to a lesser extent, in AAY002.

**Table 2:** qPCR analysis of the relative level of mRNA of BTN1 and BIT61. This represents the analysis of 3 separate experiments for the rapamycin study and 4 separate experiments for the amino acid starvation studies and each sample was run in triplicate. The error is the standard deviation. Rap: with (w) and without (w/o) rapamycin. ACT1: actin gene; PDA1: E1 alpha subunit of the pyruvate dehydrogenase.

	**Gene**	**ACT1**	**PDA1**
**Rapamycin**			

AAY002/DBY746 w/o Rap	BTN1	1.27 ± 0.22	0.96 ± 0.12
DBY746/DBY746 w Rap	BTN1	1.19 ± 0.36	1.21 ± 0.13
AAY002/DBY746 w Rap	BTN1	1.38 ± 0.23	1.19 ± 0.13
AAY002/AAY002 w Rap	BTN1	1.26 ± 0.04	1.54 ± 0.13
AAY002/DBY746 w/o Rap	BIT61	1.12 ± 0.15	0.83 ± 0.14
DBY746/DBY746 w Rap	BIT61	1.07 ± 0.04	1.00 ± 0.11
AAY002/DBY746 w Rap	BIT61	0.92 ± 0.26	0.95 ± 0.52
AAY002/AAY002 w Rap	BIT61	0.91 ± 0.04	0.89 ± 0.73

**Amino Acid Starvation**			

DBY746, 1 hr	BTN1	2.66 ± 1.10	3.30 ± 0.81
AAY002, 1 hr	BTN1	1.52 ± 0.80	2.20 ± 0.64
DBY746, 1 hr	BIT61	1.53 ± 0.68	1.66 ± 0.89
AAY002, 1 hr	BIT61	1.52 ± 0.34	1.77 ± 0.21

### HIS3 reporter strains for BTN1 expression

We developed a reporter strain to assess the role of uORFs in BTN1 gene expression and to identify genes that enhance BTN1 expression. In this setup, the expression level is determined by the transcription and translation of the reporter gene. Two strains were created with the ORF of the BTN1 gene replaced by the HIS3 gene in a his3- background. One construct retained the uORFs, while the other had the first five uORFs removed, as described earlier. The relative expression of His3p can be evaluated by growth on media lacking histidine and containing the competitive inhibitor of the HIS3 gene product, 3-amino triazole (3AT).

**Figure 4:**
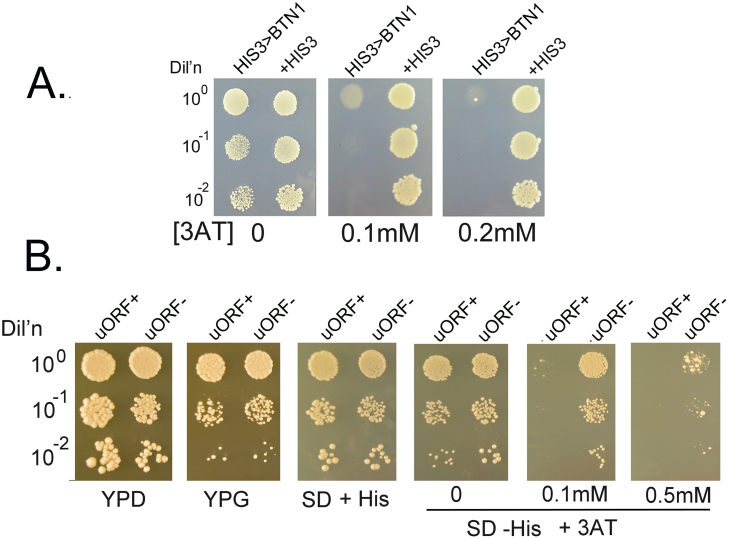
uORFs repress the expression of the HIS3 reporter. **(A)** The yeast reporter strains LWY004 (HIS3>BTN1) and LWY004 with plasmid pRS313 (+HIS3) were grown on minimal SD media lacking histidine with 3AT as indicated. **(B)** The yeast reporter strains, LWY004 (uORF+) and LWY031 (HIS3>BTN1, uORF-), were grown on the indicated media at 30 ${}^{\circ }$ C. LWY031 has converted the ATGs in the five uORFs to AAG. The parent strain is DBY746, MAT α , his3 Δ 1, leu2-3,112, ura3-52, trp1-289. LWY004 is MAT α , his3 Δ 1, leu2-3,112, ura3-52, trp1-289, btn1:: HIS3 where the HIS3 ORF replaces the ORF of BTN1. LWY031 is MAT α , his3 Δ 1, leu2-3,112, ura3-52, trp1-289, btn1::HIS3, c.-26T>A, c.-65T>A, c.-79T>A, c.-95T>A, c.-179T>A, c.-191T>A, where the HIS3 ORF replaces the BTN1 ORF and the five upstream ATGs are converted to AAG.

Figure 4 illustrates the growth of the reporter strain with and without the first five uORFs in rich media, minimal media lacking histidine, and in the presence of 3AT. Figure 4**A** compares the growth of the reporter with uORFs to the same strain converted to a HIS+ strain. The reporter strain barely grows with just 0.1 mM 3AT. Figure 4**B** shows that removing the uORFs improves cell growth, indicating that the uORFs decrease the expression of the HIS3 reporter and, in turn, the BTN1 locus. These results suggest that deleting the uORFs increases growth in the absence of histidine and at higher 3AT levels, implying that one or more uORFs lower BTN1 expression.

We used these reporter strains to identify genes that enhance the expression of the HIS3 reporter. Specifically, we transformed the HIS3 reporter strain, which lacks the uORFs, with a yeast library cloned into the vector Yep13. We selected several colonies, isolated the plasmids, and sequenced the ends using primers designed for Yep13. Two trivial genes were identified in this screen: HIS3 and ATR1, a multidrug efflux pump. Additionally, we obtained multiple clones containing tRNAs, ribosomal protein genes, and the GCN3 gene. However, the inserts were often large and included more than one gene. Indeed, the clone containing GCN3 also contained the tRNA gene for Arg. Therefore, to identify the gene responsible for induction, we subcloned the individual genes back into the Yep13 vector. We chose to subclone GCN3 and tRNA ${}^{\mathrm{Arg}}$ because expressing either of these might potentially induce the HIS3 reporter. Arginine has other connections to BTN1 and CLN3, as it has been suggested to act as a carrier for this amino acid, and defects in *CLN3*- mice have altered metabolism of arginine 28, 67, 68. The genomic library that we used was cloned into Yep13, which has the LEU2 gene as a selectable marker but also contains SUP53, which is tRNA ${}^{\mathrm{Leu}}$ (CAA). We thus cloned tRNA ${}^{\mathrm{Leu}}$ (UAA) to see if any effects with tRNA ${}^{\mathrm{Arg}}$ were specific to that tRNA.

**Figure 5:**
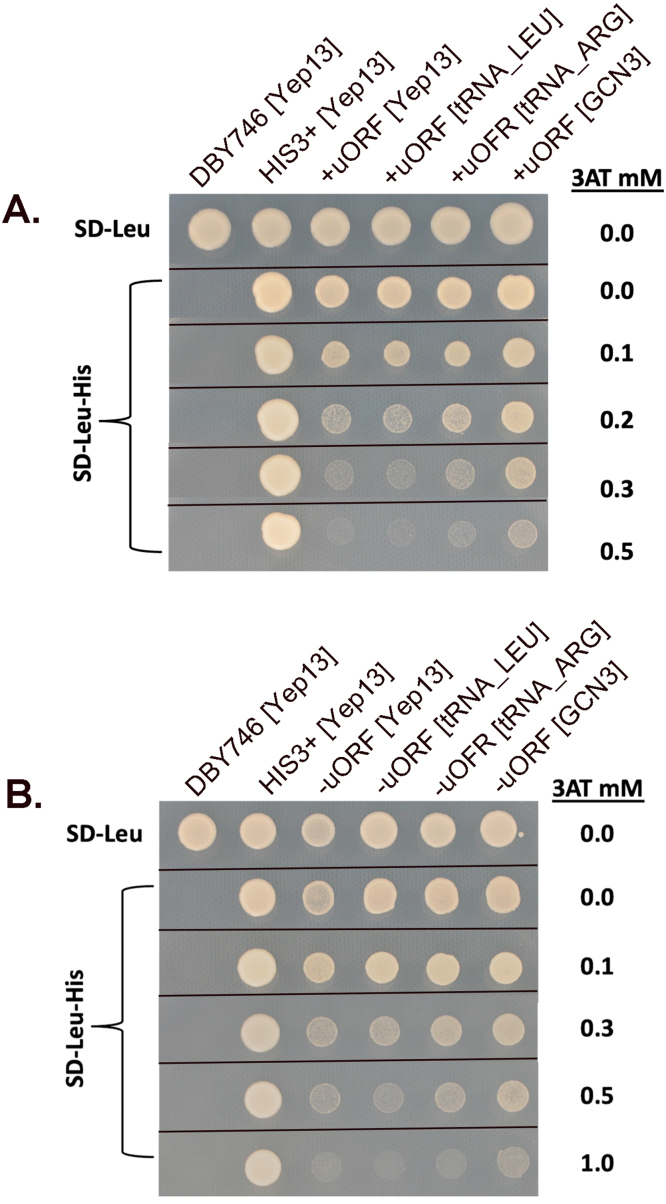
Effect of tRNA ${}^{\mathrm{Leu}}$ , tRNA ${}^{\mathrm{Arg}}$ , and GCN3 on HIS3 expression at the BTN1 locus. There are two controls: the parent strain, DBY746, and DBY746, which was converted to HIS3+ (SGY103). The cells were grown in an overnight culture, adjusted to 0.05 OD, and drop-tested in minimal media as indicated. **(A)** Expression in the reporter strain containing the uORFs after 6 days of growth at 30 ${}^{\circ }$ C. **(B)** Expression in the reporter strain minus the uORFs after 6 days of growth at 30 ${}^{\circ }$ C.

Figure 5 shows the growth of the parent strain (DBY746), the parent strain with the his3- mutation corrected (SGY103), the reporter strains, LWY004 (with uORFs) and LWY031 (without uORFs), reporter strains with and without Yep13, with tRNA ${}^{\mathrm{Arg}}$ and tRNA ${}^{\mathrm{Leu}}$ genes cloned into Yep13, and finally, with GCN3 cloned into Yep13. We added 3AT to the media to titrate the relative expression level of His3p in the cell.

There are a number of observations from this study. The presence of the plasmid with GCN3, and to a lesser extent, the plasmid with tRNA ${}^{\mathrm{Arg}}$ and tRNA ${}^{\mathrm{Leu}}$ , increased the growth of the strain compared to the parent on all media except complete media. This is much more pronounced in LWY004, the reporter strain with the uORF as compared to LWY031, which is without the uORFs. The plasmid, Yep13, which contained the SUP53 tRNA ${}^{\mathrm{Leu}}$ gene had an effect that was not increased greatly with the addition of the second tRNA ${}^{\mathrm{Leu}}$ gene. However, it is likely that the presence of tRNA ${}^{\mathrm{Leu}}$ in Yep13 had an additive effect on the induction with GCN3, possibly involving tRNA ${}^{\mathrm{Arg}}$ , but not with the additional tRNA ${}^{\mathrm{Leu}}$ gene. It is also possible that the initial clone containing GCN3 and tRNA ${}^{\mathrm{Arg}}$ induced expression of the HIS3 reporter due to the presence of both genes in the same plasmid.

These results indicate that induction primarily occurs by overcoming the inhibitory effect of one or more of the uORFs as little induction occurs when the uORFs are absent. Overall, these results suggest that the GCN pathway partially regulates the expression of the BTN1 gene via Gcn3p, and the tRNAs likely activate the pathway by increasing the levels of specific uncharged tRNAs that signal amino acid starvation 38, 39, 69.

We next looked to determine if the loss of Btn1p in the cell conferred sensitivity to amino acid starvation. In this experiment, we switched the parent strain to YPH499 as it had six auxotrophic markers, including four in amino acid biosynthetic genes. To the parent strain, we introduced the arg3 Δ and met6 Δ mutations, forming DMY938. To this strain, we then deleted BTN1, resulting in btn1 Δ , and forming DMY940. To this strain, we were able to starve the strains of the six amino acids: arginine, histidine, leucine, lysine, methionine, and tryptophan, either individually or in combination. The strain also had the ade2-101 mutation, which provided an indicator for colonies whose cells had lost their mitochondrial DNA, resulting in cytoplasmically petite ( $\rho^{o}/\rho^{-}$ ) cells. Usually, cells with the ade2 mutation turn red with the depletion of adenine due to the reduction of an intermediate formed in the adenine biosynthetic pathway by the mitochondrial electron transport chain. However, cytoplasmically petite cells lack the mitochondrial electron transport chain, and the cells remain white.

For this study, we focused on the deprivation of arginine and leucine as we tested the effect of tRNA ${}^{\mathrm{Arg}}$ and tRNA ${}^{\mathrm{Leu}}$ on the expression of the HIS3 reporter. In the first experiment, we starved the cells in media lacking arginine, leucine, and all amino acids, respectively. We found that after 48 hours at 30 ${}^{\circ }$ C, cells deprived of all amino acids exhibited a high percentage of petite mutations (around 30%), but cells starved of either arginine or leucine did not (Table 3, Figure S3). We also observed that the cells were more sensitive to amino acid starvation, regardless of which amino acid was deprived, if the cells were cytoplasmically petite and at pH 7.0.

**Table 3:** Starvation of select amino acids causes cytoplasmic petite formation. The cells were starved for 48 hrs in the absence of Leu, Arg, or all of the auxotrophic amino acids at 30 ${}^{\circ }$ C. n=3.

	**% petite**	**SD**
**DMY938, wt**		

**-Leu**	7%	3.1%
**-Arg**	2%	0.6%
**-All**	28%	7%

		

**DMY940, btn1** Δ		

**-Leu**	3%	1.1%
**-Arg**	1%	0.1%
**-All**	29%	0.2%

**Figure 6:**
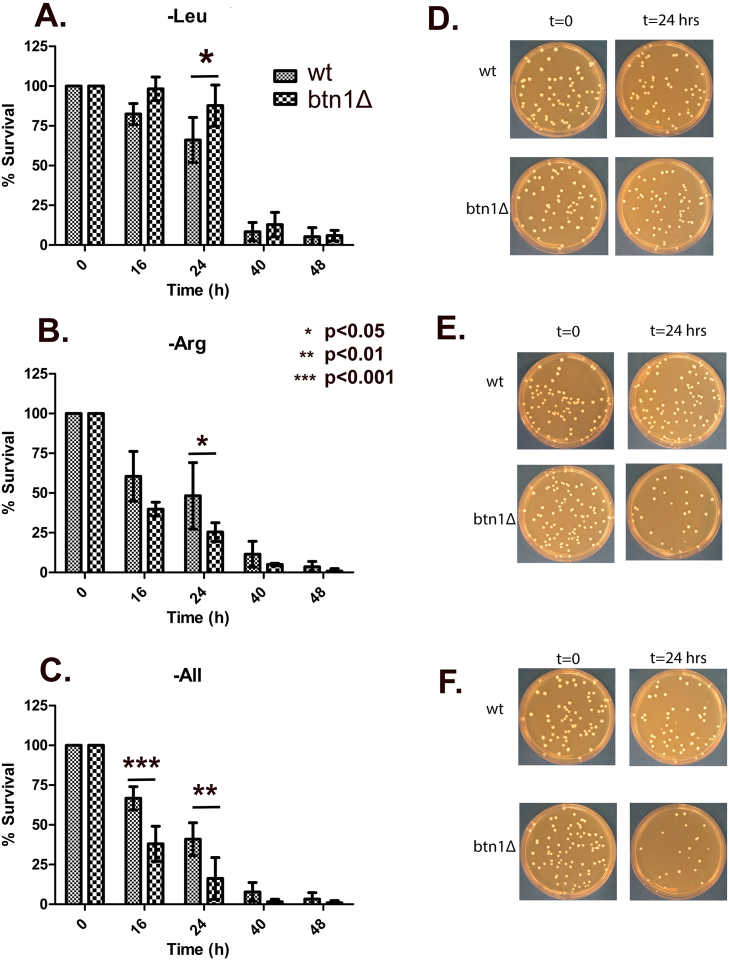
Cell Survival after Amino Acid Starvation. Cells, DMY938w and DMY940w, were starved of various amino acids: **(A, D)** leucine, **(B, E)** arginine, **(C, F)** arginine, histidine, leucine, lysine, methionine, and tryptophan (-All). The cells were cultured overnight in YPRaf. In the morning, they were washed three times with water and then transferred to starvation media at approximately 2500 cells/ml. The starvation media contained uracil, adenine, 0.2% glucose, yeast nitrogen base with 0.67% ammonium sulfate, and arginine, histidine, leucine, lysine, methionine, and tryptophan, minus the amino acid(s) being starved. After the indicated time at 30 ${}^{\circ }$ C, the cells were plated onto YPD media. (0.05 ml at t=0 and 0.075 ml at t=24 hrs) ANOVA was used to assess statistical significance.

We conducted starvation experiments at pH 7.0 using the cytoplasmically petite strains of DMY938 and DMY940, named DMY938w and DMY940w, respectively. These experiments involved simply plating cells on YPD plates and counting the resulting colonies. Figure 6 shows that starvation with all amino acids and, to a lesser extent, starvation with arginine—but not leucine—caused a higher rate of cell death in cells with the null mutation in BTN1 compared to the parent strain. Starvation on leucine seemed to have the opposite effect, with DMY940w cells surviving better than the parent strain, DMY938. These results suggest that the loss of BTN1 increases cell sensitivity to amino acid starvation, but this sensitivity depends on the specific amino acid being deprived.

## DISCUSSION

This study took a step back and, instead of asking “what is the function of CLN3?”, asked “When is *CLN3* active?”. Since the yeast BTN1 gene is homologous to the human *CLN3* gene 70, we can use yeast as a model organism to evaluate its regulation and expression related to *CLN3*. As an initial step, we reanalyzed publicly available data from 603 datasets examining yeast genome expression, focusing on those datasets where BTN1 was among the most variable genes across the different conditions yeast was exposed to. The results showed that BTN1 expression was transcriptionally regulated in response to stress conditions, including amino acid starvation, exposure to rapamycin and arsenic, oxidative stress, sporulation conditions, and heat shock (Table 1). These results are consistent with what is known about the effect of loss of CLN3 in various tissues and organisms. First, CLN3 has been reported to be important for stress granule dynamics in HeLa cells 71, altered the response to oxidative stress in *Drosophila* 72, and reactive oxygen species were increased in JNCL patient fibroblasts 73.

Second, arsenic is a phosphate mimic and uncouples ATP synthesis from electron transport, resulting in lower ATP production and superoxide formation 74. In addition to the relationship of oxidative stress to CLN3, defects in CLN3 have been reported to impair oxidative phosphorylation. *CLN3* Δ HeLa cells were shown to have lower extracellular acidification rates and oxygen consumption rates as compared to wild-type HeLa cells 71, suggesting slower metabolism. Fibroblasts from patients showed lower levels of the mitochondrial ATP synthase as measured by mitochondrial ATPase activity 75 and lower cellular levels of ATP 76. Using RPE-1 retinal cells, extracellular acidification rates were lower and showed reduced sensitivity to the ATP synthase inhibitor, oligomycin, when the cells were treated with siRNA against exon 8 of *CLN3* as compared to the control cells 77.

Third, in yeast *Schizosaccharomyces pombe*, btn1 Δ causes temperature sensitivity, which is rescued by 2M sorbitol 78. The same study also showed increased sensitivity to nitrogen starvation, and overall concluded that TOR signaling is involved with CLN3. This is consistent with increased expression under heat shock conditions, during sporulation, with low nitrogen availability, and changes in osmolarity, including 0.4 M NaCl. Overall, the conditions that induce transcription of BTN1 are consistent with the phenotypes associated with defects in BTN1 and *CLN3*.

### Transcriptional initiation and termination

The 5’ RACE results indicated multiple 5’ ends of the mRNA with clusters around −24, −32, −39, and −60 (Figure 3). Multiple initiation sites from a single promoter are common in yeast 79, 80, but the range suggests that there are multiple promoters. DNA sequence analysis of the upstream region shows TATAA boxes at −164, −200, and −211, which would place transcriptional initiation about 40 bp downstream or around −120, closest to the coding frame of BTN1. However, yeast often has non-TATA box promoters. Additionally, there are multiple motifs for known transcription factors in front of BTN1, which are also present in front of the BTN1 homolog in yeast *Schizosaccharomyces pombe*. 

The most abundant motif is that for Fkh1p and Fkh2p, which are located at −169, −163, −161, −157, −65, −51, −35, and −10. Fkh1p and Fkh2p are Forkhead family of transcription factors; the human homologs are *FOXJ3*, *FOXK1* and *FOXK2*. Fkh1p and Fkh2p are involved in the cell cycle and deletion of both causes pseudohyphal growth 81. Pseudohyphae occur in yeast when the cells elongate but fail to separate after division.

There are binding motifs for Hsf1p at −150 and −151. Hsf1p is a heat shock transcription factor involved in the expression of the ribosome quality control complex (RQC), which includes an E3 ubiquitin ligase 82. HSF1 is induced during various stress conditions including heat shock 83, oxidative stress 84, and glucose starvation 85. The human homologs are HSF1-3, which are associated with neurodegenerative diseases.

There is a binding motif for several transcription factors, Yap1p, Cad1p, Yap3p, Cin5p, and Yap5p, at −72. These genes are all involved in the expression of genes during oxidative stress 86. There is no apparent human homolog for these genes.

Rtg3p is involved in the transcription of genes involved in retrograde movement and the TOR pathway and is expressed under stress 87. There is an Rtg3p binding motif at −97. *CLN3* has been implicated in a role in trafficking 21, and we and others 78 are suggesting the TORC1 pathway. The human homolog of Rtg3p is MITF (melanocyte-inducing transcription factor).

Overall, these putative transcription binding site motifs are consistent with the conditions that induce the expression of BTN1. The multiple binding sites for Fhk1p and Fhk2p suggest that BTN1 may also be involved in the cell cycle. Our studies were limited to the stress condition of amino acid starvation and, related to that, inhibition with rapamycin. New transcription sites may emerge under various stress conditions, including oxidative stress and heat shock, such that the uORFs will be relevant to the expression of BTN1.

### Translation regulation of BTN1

The reporter constructs indicate that one or more of the uORFs decrease the translation of the mRNA. The increase in reporter expression with the expression of GCN3 supports the hypothesis that BTN1 is under translational control. Based on our 5’ RACE results, only uORFs at −27 and possibly −66 could affect the translation of the mRNA. However, another study has identified −90 at the 5’ end of BTN1, suggesting that the transcriptional initiation site is subject to change 65.

The expression of the HIS3 reporter indicates the activity of the BTN1 locus. The yeast reporter strain LWY004 has the HIS3 coding sequence inserted, replacing the ORF of BTN1. The entire BTN1 ORF is replaced, except for the first seven codons, which are retained to preserve their effect on translation. The reporter strain can grow without histidine in the media, but only faintly with just 0.1 mM 3AT. In contrast, strain SGY103, which shares the same parent as LWY004 but is HIS3+, can grow on more than 10 mM 3AT. Therefore, the expression of HIS3 at the BTN1 locus is much lower compared to its expression at the HIS3 locus.

To identify the genes that induce the expression of the HIS3 reporter, we used the strain without the uORFs, LWY031. This method was used to find genes that either increase the transcription or translation of the reporter. Selection was done at 1 mM 3AT, which is well above the concentration needed to ensure strong positive selection. Because the plasmid library’s inserts were large, it was necessary to subclone the genes present in positive clones to determine which gene was affecting reporter expression. However, there are two caveats to this approach. First, we selected transformants on media without leucine and histidine (the selective marker in Yep13) and added 1 mM 3AT to target the desired genes. However, this method allowed any single transformant to contain multiple plasmids with different inserts. As a result, the selection might favor more than one plasmid that work together to increase reporter expression. This could explain why growth on 3AT was reduced compared to the conditions during clone isolation. The second caveat is that two genes within the same plasmid might be working together. Indeed, GCN3 is located very close to tRNA ${}^{\mathrm{Arg}}$ , and both genes were shown to induce HIS3 reporter expression.

The cumulative effect of expressing multiple genes on the activation of the HIS3 reporter is anticipated. Since each pathway includes several genes, over-expressing just one gene in that pathway typically has a minor effect. However, in this study, even a small effect is still sufficiently significant to identify genes involved in regulating the target gene. This is partly enabled by the fact that the selection scheme allows multiple plasmids within a single transformant.

Another factor in this selection scheme is the growth conditions. Using 3AT in a his3-strain, we deprive the cells of histidine. Surprisingly, despite this, the growth of the reporter strains, LWY004 (with uORF) and LWY031 (without uORF), was not strong. Therefore, the loss of histidine was not enough to trigger the expression of the reporters, whether or not they contained uORFs. This contrasts with the early study on GCN4, which showed that 3AT induced the expression of GCN4, HIS4, and HIS3 88. Also, Gcn2p is activated by tRNA ${}^{\mathrm{His}}$ 38, suggesting that the pathway that induces BTN1 is distinct from that which induces GCN4.

### Amino acid starvation and cell viability

Several variables were considered when determining the conditions used to evaluate yeast viability during amino acid starvation. First, the specific amino acid being starved can be critical because it affects the outcome. For example, we know that tRNA ${}^{\mathrm{Arg}}$ , and to a lesser extent tRNA ${}^{\mathrm{Leu}}$ , can induce the expression of the HIS3 reporter. Therefore, starvation of arginine can yield different results than starvation of leucine. Second, the pH of the medium can be significant. Previous studies have shown that vacuolar and lysosomal pH are altered in yeast or human cells defective in BTN1/*CLN3* 73, 89, 90. In yeast, a defect in BTN1 can be partly compensated for by using an acidic medium, similar to how defects in the vacuolar ATPase can be corrected 91. Third, there is evidence that defects in *CLN3* affect mitochondria and oxidative phosphorylation 71, 75–77. Additionally, BTN1 in yeast is induced two-fold during the diauxic shift from glucose to a nonfermenting carbon source, which increases the involvement of oxidative phosphorylation 92. As a result, a *CLN3* defect can have a cumulative effect, combining issues in oxidative phosphorylation with other defects. Using yeast, we can examine these effects because we can make the cells auxotrophic for any amino acid, control the pH of the medium, and use cytoplasmically petite strains that are defective in oxidative phosphorylation.

We modified YPH499 by adding auxotrophic markers of arg3 Δ and met6 Δ . The ade2-mutation allows us to identify cytoplasmically petite strains by their color; they turn white after adenine depletion in the media. We choose to deplete arginine and leucine separately from the media and then all amino acids, which limits histidine, leucine, lysine, methionine, and tryptophan. Surprisingly, starvation with all amino acids caused cells to become cytoplasmically petite; however, this did not occur with deprivation of either arginine or leucine alone. The percentage of petites increased as the duration of starvation lengthened. We also know that starvation of a single amino acid alone is enough to trigger cytoplasmic petite formation. However, the formation of cytoplasmic petites was not impacted by the btn1 Δ mutation under the tested conditions. Currently, the mechanism behind cytoplasmic petite formation in these conditions remains unknown. This situation is unique because the cells are not dividing, so large deletions in the multiple copies of mtDNA must occur without cell division. One possible mechanism involves the production of reactive oxygen species that damage the mtDNA.

The second result showed that using cytoplasmic petite strains, the presence of the btn1 Δ mutation increased lethality during starvation conditions that deplete all amino acids and, to a lesser extent, arginine. Interestingly, leucine starvation had a weak but apparent protective effect on cellular viability. These findings are intriguing because they relate to aspects of regulation and protection. A key question is whether human cells defective in *CLN3* respond similarly to amino acid starvation and if amino acid supplements can help reduce some issues related to juvenile Batten disease.

Yeast offers many advantages over human cell lines for the study of Batten disease, including rapid screening strategies for mutations and genes, a short generation time, a smaller genome, and the ability to produce large amounts of protein for biochemical and biophysical studies. There are 13 different genes that, when defective, are reported to cause Batten disease 1, 7–10. Of these, 5 have yeast homologs, suggesting that the pathway responsible for these genes is a basic pathway conserved in yeast. However, yeast is still only a model system, and results from all model systems must be translated to the mammalian system, as there are certainly differences.

The regulatory mechanisms identified in yeast and subsequently verified in mammalian systems might provide therapeutic strategies. For instance, we might uncover genes that, when down- or up-regulated, can offset some of the problems associated with the loss of CLN3 function. Or we might be able to avoid physiological states that rely on CLN3, as patients with phenylketonuria avoid phenylalanine in their diet. While this is unlikely to completely treat all of the issues associated with Batten disease, it might be one of many therapeutic strategies that, when combined, result in a manageable lifestyle.

## MATERIALS AND METHODS

All the media and chemicals were purchased from either United States Biologicals or VWR International. The molecular biology enzymes and kits were from New England Biolabs, USA. All oligonucleotides were purchased from Integrated DNA Technologies, Inc., USA, and double-stranded DNA was obtained from Twist Biosciences, San Francisco, CA. All the plasmids, primers, and synthetic dsDNA used in this study are listed in Table S2.

### Media

The following media were used in this study: YPD, containing 1% yeast extract, 2% bactopeptone, and 2% glucose; YPG, with 1% yeast extract, 2% peptone, and 3% glycerol (v/v); YPRaf, with 1% yeast extract, 2% peptone, and 2% raffinose; YPGal, with 1% yeast extract, 2% peptone, and 2% galactose; SD, containing 0.17% yeast nitrogen base, 0.5% ammonium sulfate, and 2% glucose; and, when indicated, supplements such as 20 mg/l adenine, uracil, L-tryptophan, L-histidine, L-arginine, 30 mg/l L-leucine, L-lysine, and 40 mg/l D,L-methionine. Starvation media S1 and S2 are based on SD media, with S1 including 20 mg/l adenine and 20 mg/l uracil. S2 consists of 0.67% yeast nitrogen base with ammonium sulfate, 20mg/ml adenine, 20 mg/l uracil, 0.2% glucose, and 10 mM HEPES buffer at pH 7.0.

**Table 4:** Strains used in this study .

	**Parent/Source**	**Genotype**
DBY746	D. Botstein 93	MAT α , his3 Δ 1, leu2-3,112, ura3-52, trp1-289

DMY929	YPH499	MAT a, ade2-101, his3- Δ 200, leu2- Δ 1, lys2-801, trp1- Δ 63, ura3-52, btn1:: eGFP

AAY002	DBY746	MAT α , his3 Δ 1, leu2-3,112, ura3-52, trp1-289, c.-26T>A, c.-65T>A, c.-79T>A, c.-95T>A, c.-179T>A, c.-191T>A, c.90T>G, c.93T>A, c.99C>T

SGY103	DBY746	MAT α , leu2-3,112, ura3-52, trp1-289

LWY004	DBY746	MAT α , his3 Δ 1, leu2-3,112, ura3-52, trp1-289, btn1:: HIS3

LWY005	LWY004	MAT α , his3 Δ 1, leu2-3,112, ura3-52, trp1-289, btn1:: HIS3 [pRS313]

LWY031	DBY746	MAT α , his3 Δ 1, leu2-3,112, ura3-52, trp1-289, btn1::HIS3, c.-26T>A, c.-65T>A, c.-79T>A, c.-95T>A, c.-179T>A, c.-191T>A

SGY118	SGY103	+ [Yep13], (ATCC:37323)

SGY119	DBY746	+ [Yep13]

SGY108	LWY004	+ [Yep13]

SGY116	LWY004	+ [pGCN3]

SGY112	LWY004	+ [p_tRNA ${}^{\mathrm{Leu}}$ ]

SGY121	LWY004	+ [p_tRNA ${}^{\mathrm{Arg}}$ ]

SGY109	LWY031	+ [Yep13]

SGY125	LWY031	+ [p_tRNA ${}^{\mathrm{Arg}}$ ]

SGY115	LWY031	+ [pGCN3]

SGY124	LWY031	+ [p_tRNA ${}^{\mathrm{Leu}}$ ]

YHP499	P. Hieter 94	MAT a, ade2-101, his3- Δ 200, leu2- Δ 1, lys2-801, trp1- Δ 63, ura3-52

DMY938	YPH499	MAT a, ade2-101, his3- Δ 200, leu2- Δ 1, lys2-801, trp1- Δ 63, ura3-52, arg3 Δ ::loxP, met6 Δ ::loxP, btn1::KanR

DMY940	YPH499	MAT a, ade2-101, his3- Δ 200, leu2- Δ 1, lys2-801, trp1- Δ 63, ura3-52, arg3 Δ ::loxP, met6::loxP,

DMY938w	YPH499	MAT a, ade2-101, his3- Δ 200, leu2- Δ 1, lys2-801, trp1- Δ 63, ura3-52, arg3 Δ ::loxP, met6::loxP, btn1::KanR, ρ ${}^{0}$ / ρ ${}^{-}$

DMY940w	YPH499	MAT a, ade2-101, his3- Δ 200, leu2- Δ 1, lys2-801, trp1- Δ 63, ura3-52, arg3 Δ , met6 :loxP, btn1::KanR, ρ ${}^{0}$ / ρ ${}^{-}$

Notes: btn1::eGFP and btn1::HIS3 retained 1 st 7 codons of the BTN11/YHC3 gene to preserve translational control. Three silent mutations in AAY002 to prevent recutting at gRNA site. The eGFP coding sequence has been codon optimized for expression in yeast.

### Yeast strains

The list of yeast strains Table 4. For strain development, the plasmid, p414-TEF1p-Cas9-CYC1t was a gift from George Church 95 (Addgene plasmid # 43802; http://n2t.net/addgene:43802; RRID:Addgene_43802) and plasmid, p426-SNR52p-gRNA.CAN1.Y-SUP4t was also a gift from George Church (Addgene plasmid # 43803; http://n2t.net/addgene:43803; RRID:Addgene_43803). For the development of p426-SNR52p-gRNA.YHC3.Y-SUP4t, p426-SNR52p-gRNA.CAN1.Y-SUP4t was digested with ClaI and the cut plasmid was transformed into yeast along with DNA fragment that encoded the gRNA for BTN1/YHC3 gene and which spanned over the gRNA for the CAN1 gene creating the new plasmid by gap repair 96. The knock-out yeast constructs were made and verified as described 97 using the primers listed in Table S2.

tRNA ${}^{\mathrm{Arg}}$ YNCE0028C, (ACG), decodes CGU, CGC, and likely CGA. The HIS3 gene has 11 arginine codons: 1 CGA and 10 AGA. AGA is the most common arginine codon used in *S. cerevisiae*, followed by AGG. tRNA ${}^{\mathrm{Leu}}$ , YNCN0020C, (UAA), decodes UUA. There are 24 alanine codons in HIS3, with 16 being UUA. UUA and UUG are the most commonly used alanine codons in yeast *S. cerevisiae.* The SUP53 gene on Yep13, tRNA ${}^{\mathrm{Leu}}$ (CAA), decodes UUG. There are 4 alanine codons, UUG, in HIS3.

### Gene expression analysis

Microarray data for gene expression was obtained from the Saccharomyces Genome Database (SGD) 47 at http://sgd-archive.yeastgenome.org/expression/microarray/ on May 23, 2023. A Python script utilizing NumPy and Pandas was developed to read, extract, and analyze data from the downloaded microarray datasets. We excluded any datasets with fewer than 4000 genes. Datasets with irregular expression levels (fold-change values > 100 across different conditions) were also excluded, as well as any datasets lacking expression data for BTN1. To allow comparison across all remaining datasets, the expression levels of BTN1 (YJL059w) were normalized as the average relative to ACT1 (YFL039c) and PDA1 (YER178w), both housekeeping genes. This normalization should ensure consistent gene expression regardless of conditions, allowing us to compare BTN1 expression across different datasets. The datasets were sorted based on these relative expression values. The 15 datasets with the highest variance were chosen for further analysis. We examined the fold change of BTN1 at its highest level in each study compared to the baseline expression of BTN1 in the control, as reported in Table 1. To identify genes regulated similarly to BTN1 across the dataset conditions, we used k-means clustering (Python package sklearn) to group the genes into 16 clusters. We then used Enrichr 98, 99 to find gene ontologies related to biological processes that were enriched with genes appearing in the same cluster as BTN1 in at least four datasets. We also examined which gene ontology biological processes were enriched in each study and which were common across all studies.

### Culturing Yeast and total RNA isolation

*S. cerevisiae* strains, DBY746 (wt) and AAY002, were grown as a preculture from a single colony in YPD media (3 ml) at 30 ${}^{\circ }$ C. The test culture was inoculated into YPD media (40 ml) until the cell concentration reached 1 × 10 ${}^{7}$ /ml. For rapamycin treatment, rapamycin (100 nM) was added, and incubation was continued for 30 min at 30 ${}^{\circ }$ C 62. For amino acid starvation, the cells were harvested at 3000 × g at 4 ${}^{\circ }$ C, washed twice in 5 mL of ice-cold sterile water. Washed cells were resuspended in amino acid starvation media S1 and cultured in 40 mL of amino acid starvation media for 1 hour at 30 ${}^{\circ }$ C. After treatment with rapamycin or amino acid starvation media, the cultures were cooled on ice, and cells were collected by centrifugation at 3000 × g for 5 min at 4 ${}^{\circ }$ C. The cells were transferred to a sterile 2 mL screw-cap tube, centrifuged again at 3000 × g for 5 min at 4 ${}^{\circ }$ C, and the pellet was stored at −80 ${}^{\circ }$ C until use.

### RNA Isolation

We used a traditional method to extract RNA from yeast cells because there was a report indicating that mRNA response to stress is resistant to extraction using the hot phenol method 100. All reagents and supplies used after phenol extraction were free of RNase. The yeast pellets were thawed on ice, and 0.3 g of 0.5 mm glass beads were added along with 0.4 mL of TRIzol (Ambion, 15596018). The cells were broken using a BeadBeater (Mini BeadBeater 16 Disruptor, Biospec Products, Bartlesville, OK) at 4 ${}^{\circ }$ C with 1-minute intervals followed by 1-minute cooling, for a total of 9 minutes. Chloroform (80 μ L) was added to the lysed cells and incubated at room temperature for 2–15 minutes, then centrifuged at 10,000 xg for 30 seconds. The supernatant was collected and centrifuged again at 20,000 x g in the cold room for 10 minutes. The supernatant was transferred and extracted with an equal volume of phenol/chloroform and isoamyl alcohol (50/49/1) pH 4.3. The mixture was centrifuged at 20,000 x g in the cold room for 5 minutes, and the aqueous layer was collected. Sodium acetate (0.3 M, pH 5.5) and two volumes of ethanol were added to precipitate the RNA. The RNA was pelleted by centrifugation at 10,000 xg for 10 minutes, then washed with 66% ethanol. The pellet was lightly dried *in vacuo* and dissolved in RNase-free water (0.2 mL). The RNA concentration was measured using a NanoDrop spectrophotometer (ThermoFisher Scientific) at 260 nm.

Poly-A RNA was isolated from a total of 80 μ g of RNA using oligo d(T)25 magnetic beads (New England Biolabs, 61002), following the protocol and kit purchased from New England Biolabs (S1550). The RNA was eluted at 50 ${}^{\circ }$ C with 100 μ L of elution buffer (20 mM Tris-HCl, pH 7.5, 1 mM EDTA), its concentration was measured as described above, and the RNA was stored at −80 ${}^{\circ }$ C.

### cDNA synthesis

Poly-A RNA (0.26 μ g) was treated with DNAse I (0.2 U, New England Biolabs M0303) for 10 minutes and then stopped at 75 ${}^{\circ }$ C for 15 minutes. Primer, oligo dT/random hexamer mix (10 μ M/12 μ M), was added to the RNA, heated to 65 ${}^{\circ }$ C, and then placed on ice. The solution (10 μ L) was added to reaction buffer (10 μ L), AMV reverse transcriptase (10 U) (New England Biolabs, M0277), and murine RNase inhibitor, 8 U (New England Biolabs, M0314). The reaction was heated to 25 ${}^{\circ }$ C for 5 minutes, then to 42 ${}^{\circ }$ C for 1 hour, and finally to 85 ${}^{\circ }$ C for 5 minutes. To this, RNase H (1 U, New England Biolabs, M0297) was added and incubated for 20 minutes at 37 ${}^{\circ }$ C, then for 20 minutes at 65 ${}^{\circ }$ C.

qPCR was performed using the TaqMan protocol with OneTaq Hot Start DNA polymerase (New England Biolabs, M0481) and the standard reaction buffer (New England Biolabs, B9022) along with ROX reference dye (Invitrogen, 54881). The primers used (Table S2) included the FAM Reporter with NFQ-MGB quencher (Integrated DNA Technology, Coralville, Iowa). The QuantStudio ViiA 7 Real-Time PCR system (ThermoFisher Scientific) was operated in the Fast 96-well (0.1 mL) setting to obtain the ΔΔ CT values.

### 5’ and 3’ RACE

5’ RACE was performed using two methods. **A.** The first method was the traditional approach using polyA RNA, followed by cDNA synthesis, then terminal transferase with dATP, second strand synthesis, and PCR amplification. **1.** 25 ng of polyA RNA and 10 μ M template-specific primer were combined with 10 μ M dNTPs. The reaction (10 μ L) was heated to 65 ${}^{\circ }$ C for 5 minutes and then cooled on ice. **2.** AMV reverse transcriptase buffer, 8 units of murine RNase inhibitor (New England Biolabs, M0314), and 1 unit of AMV reverse transcriptase (New England Biolabs, M0277) were added, then incubated at 42 ${}^{\circ }$ C for 1 hour, followed by 85 ${}^{\circ }$ C for 5 minutes. **3.** The DNA was purified using SPRIselect magnetic beads (60 μ L) (Beckman Coulter) per reaction according to the manufacturer’s protocol. The cDNA was eluted with water (20 μ L). **4.** Terminal transferase buffer (Roche, 11243276001), 34 μ M CoCl ${}_{2}$ , 27 μ M dATP, and 120 units of terminal transferase (Roche, 03333566001) were added to a final volume of 75 μ l. The sample was incubated for 1.5 hours at 37 ${}^{\circ }$ C, then heated to 80 ${}^{\circ }$ C for 3 minutes. **5.** From this reaction, 5 μ L was taken and added to a 50 μ L reaction containing 1X OneTaq DNA polymerase buffer, 1.25 units of OneTaq (New England Biolabs, M0480), and 0.324 μ M QT-RACE primer. The reaction was run at 94 ${}^{\circ }$ C for 5 minutes, then at 37 ${}^{\circ }$ C for 30 minutes, 50 ${}^{\circ }$ C for 5 minutes, 68 ${}^{\circ }$ C for 40 minutes, and then stored or further processed. The sample was diluted 1:500 in sterile water. **6.** An aliquot (1 μ L) of this dilution was added to a 50 μ L reaction mixture containing TaqDog Hot Start DNA polymerase and reaction buffer (HTQDG050, Bulldog Bio), 0.5 μ M Qo-RACE and Po primers. The PCR was performed with cycles of 94 ${}^{\circ }$ C for 30 seconds, 55 ${}^{\circ }$ C for 30 seconds, 72 ${}^{\circ }$ C for 30 seconds, repeated 35 times, followed by a final extension at 72 ${}^{\circ }$ C for 5 minutes.

**B.** The second method we used for 5’ RACE used the template switching method following the manufacturer’s suggested protocol (NEB #M0466). Total RNA (0.5 μ g) and primers BIT61_319.rev.pri/BTN1_365.rev.pri (1 μ M) and dNTPs (1 mM) were used for the primary cDNA synthesis. The second strand was synthesized using the template switching oligonucleotide (TSO), and the products were amplified with primers BTN1_312_a.rev.pri/BIT61_217.rev.pri and TSO.pri, using Q5 high-fidelity DNA polymerase (NEB, M0491).

### 3’ RACE

From the cDNA reaction, 1 μ L is added to a 25 μ L reaction containing 0.5 μ M Qo-RACE and Po primers, and 1x TaqDog Hotstart enzyme and reaction mix. This is run at 94 ${}^{\circ }$ C, 30 s, 55 ${}^{\circ }$ C, 30 s, 72 ${}^{\circ }$ C, 30 s, repeated for 35 cycles, followed by 72 ${}^{\circ }$ C for 5 min. This reaction is diluted 1:200 in water and 1 μ l is added to another PCR reaction as above but containing 0.5 μ M Qi-RACE and Pi primers.

We used poly A RNA and treated with DNase (New England Biolabs, M0303S) treated total RNA. AMV reverse transcriptase (New England Biolabs, M0277L) and RNase H (New England Biolabs M0297) were used to make the cDNA.

### 5’ RACE-NGS data analysis

The 5’ RACE PCR products from method A were PCR purified and tagged with adapter sequence, normalized and sent to Novogene, USA for NGS on NovaSeq X plus. The data was processed using Galaxy 101 web platform at https://usegalaxy.org/. Cutadapt 102 was used to filter the sequences with at least the last 8 bases of the adapter sequence. Bowtie2 103 was used to map the sequencing reads to corresponding reference genomes of DBY746 (*S. cerevisiae* S288C genome assembly R64 (sacCer3)) and AAY002 (mutated the upstream open reading frames to match sequence of the reads). The .bam files generated from Bowtie2 were converted to .bed files using BEDTools 104 package (Bjoern A. Gruening (2014), Galaxy wrapper). Downloaded the .bam and .bed files to a local computer for further analysis. Python (https://www.python.org/) code was written to determine Transcription Start Site (TSS) position with respect to the start codon and to export the positions to a text file. Using code written in R programming language (https://www.r-project.org/; https://posit.co/products/open-source/rstudio/) a matrix was created with TSS position and corresponding counts for each study, normalized the data as counts per million using Trimmed Mean of the M-values method with Bioconductor package edgeR 105, 106 v.4.4.0. Converted the counts into log10 of the percentage of the count values and generated a heat map of the counts using pheatmaps 107 (pretty heatmaps v.1.0.12) package in R.

### Identification of Genes that activate expression of the HIS3 reporter

Yeast strain, LWY031, was transformed with a yeast genomic library (ATCC 37323, Yep13 based) and colonies were selected on minimal media without leucine and histidine and in the presence of 3-aminotriazole (1 mM). Colonies that grew were selected and grown in liquid minimal medium without leucine. Plasmid DNA was extracted from the cells and transformed into bacteria, NEB5 α (New England Biolabs, C2987), and selected on LB medium with ampicillin. The plasmid DNA was isolated from the bacteria, and the DNA was sequenced using Yep13 primers, 325.pri and 519.rev.pri, which flanked the BamHI site. The clone typically was large enough to include multiple genes. As such, the clone was reduced to a single gene by PCR and recloning into Yep13. This subclone was transformed into the tester strains.

### Amino acid starvation

Yeast was grown in an overnight preculture in YPRaf media (3 mL) to mid-log stage. The cells (1 mL) were harvested and washed three times with water, or two times with S2 media, added to S2 media at a target concentration of 2500 cells/mL and rotated at 30 ${}^{\circ }$ C. After 24 hours, the cells were spread onto YPD plates, and incubated at 30 ${}^{\circ }$ C for 2–4 days. The volume of the cells plated depended on the time point: 0.05 ml (t=0), 0.075 mL (t=16 and 24 hrs), 0.10 mL (t=40 and 48 hrs), and water was added to a total of 0.2 mL. The experiment was done in triplicate with 3 unique freshly grown colonies. Cell death was based on the number of colonies relative to the first time point.

## SUPPLEMENTAL MATERIAL

All supplemental data for this article are available online at http://microbialcell.com/researcharticles/2026a-pillalamarri-microbial-cell.

.

## CONFLICT OF INTEREST

The authors declare no conflict of interest.

## ABBREVIATIONS

GCD – general control derepressible genes

NCL – neuronal ceroid lipofuscinoses

uORF – upstream open reading frame
