# Supplementary material for: TOR-dependent regulation of the yeast homolog of the juvenile Batten Disease-associated gene CLN3
Source: Microb Cell. 2026 Mar 11;13:131–47. doi: 10.15698/mic2026.03.872 (PMC13093828; doi:10.15698/mic2026.03.872)
Supplement: Supplementary file 1 — . [file mic-13-131-s01.pdf]

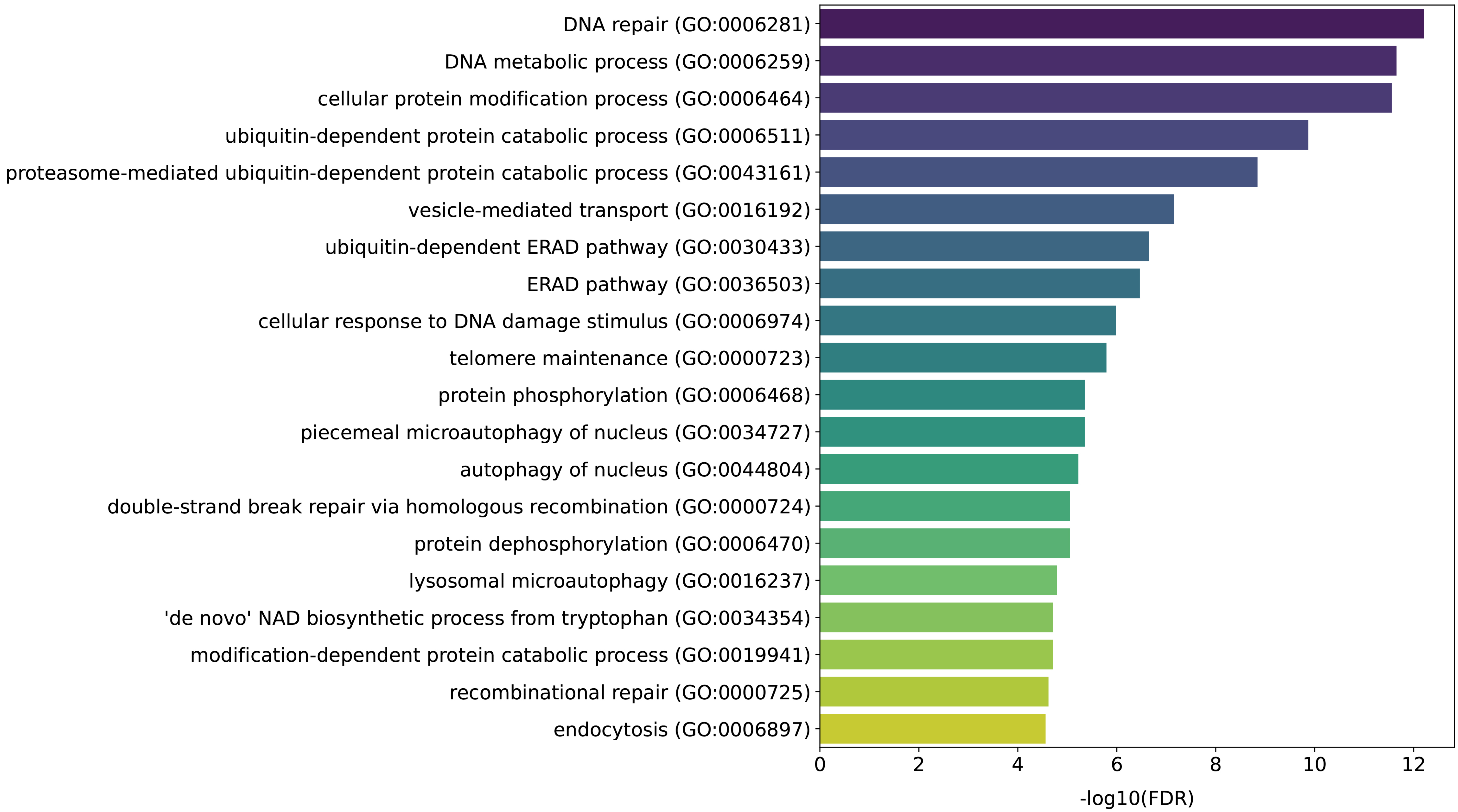

Figure 1S: Gene Ontology (GO) analysis of the 15 datasets with the most variable expression of BTN1. Most enriched pathways involve DNA metabolism, repair, or protein modifications and degradation and transport. The X-axis displays the -log10 values of the False Discovery Rate (FDR) for the pathways.

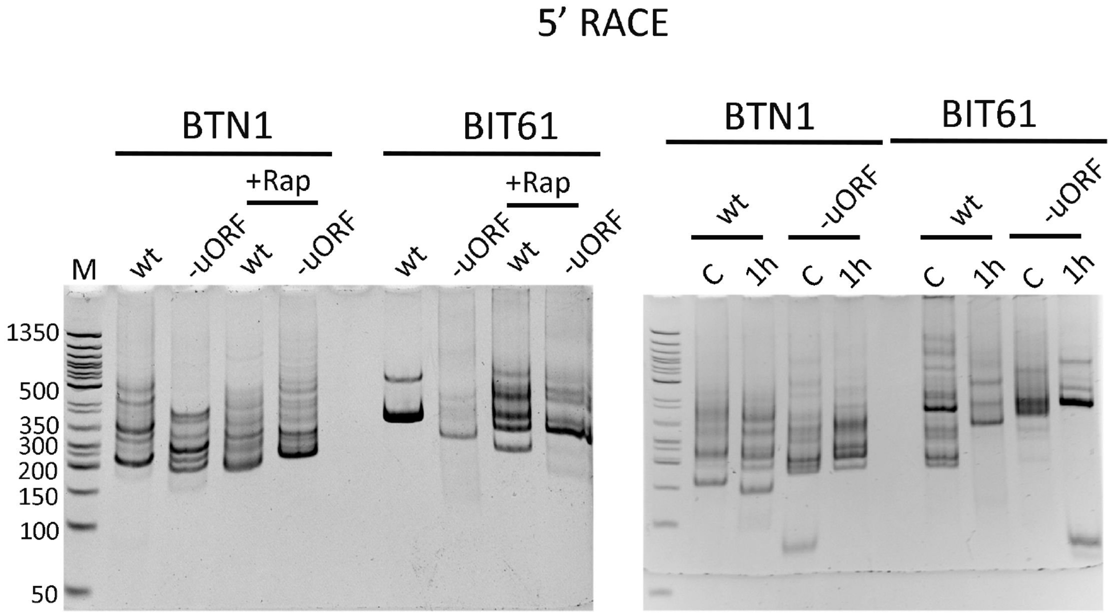

Figure 2S. 5' RACE products with rapamycin treatment and amino acid starvation.

The cells from strains DBY746 (wt) and AAY002 (-uORFs) were treated as described in Materials and Methods. The poly-A RNA was used to perform 5' RACE using the traditional method, as described in Materials and Methods. The products were amplified with Qi primers, at 99 for BTN1 and 217 for BIT61. The products were separated on a 4% polyacrylamide gel and stained with ethidium bromide.

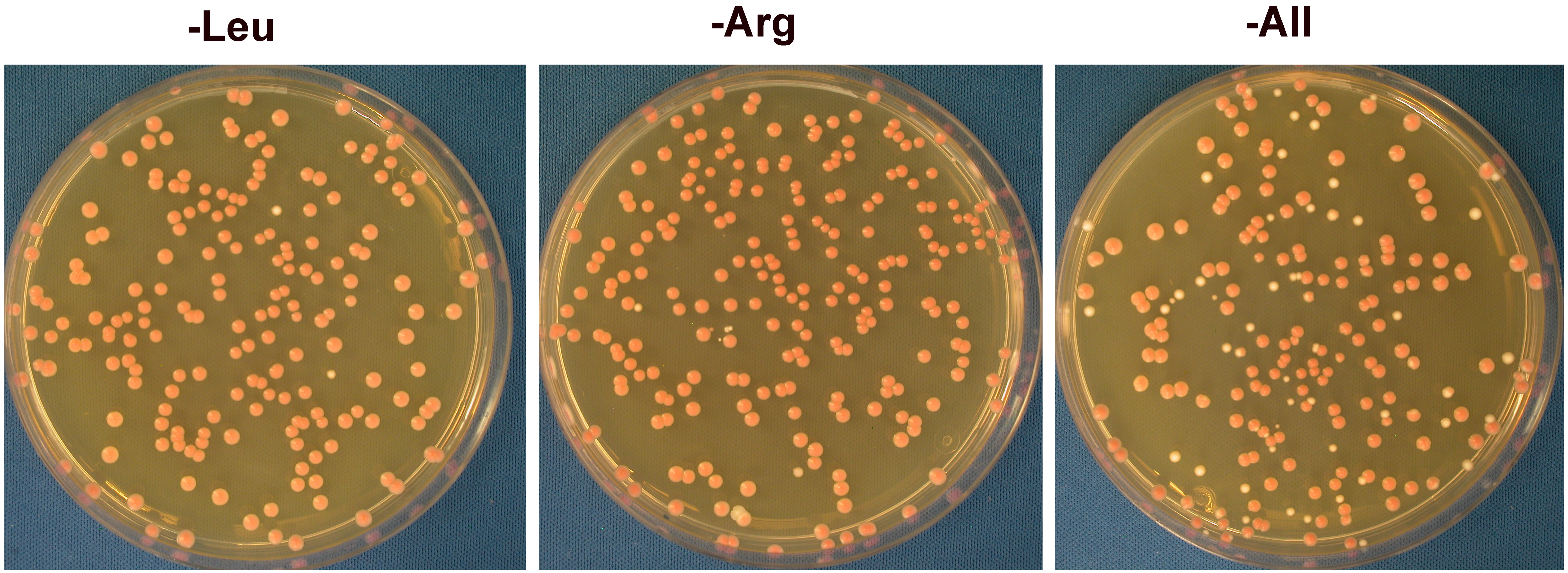

Figure 3S. Formation of cytoplasmic petite mutations due to amino acid starvation. The cells, DMY940 (BTN1Δ), were starved for 48 hours at 30°C in the absence of the amino acids arginine, histidine, leucine, lysine, methionine, and tryptophan. A preculture of the cells was grown overnight in YPRaf medium at 30°C. In the morning, they were washed three times with water and then transferred to starvation medium at a concentration of approximately 500 cells/ml. The starvation media contained uracil, adenine, 0.2% glucose, and 0.67% yeast nitrogen base with ammonium sulfate.

Table 1S: Pathways enriched in each of the 15 studies where BTN1 expression varies the most. The header row displays the author, publication year, and GEO ID (if available), along with relevant references. The index column lists selected GO terms that were found to be enriched and are discussed in the manuscript. The total count indicates the number of datasets in which a specific biological process (GO term) is either enriched (Y) or not enriched (N).

	Oromendia_2012_GSE40073 (1)	Hardwick_1999 (2)	Cheung_2008_GSE12272 (3)	Roy_2013_GSE38478 (4)	Thorsen_2007_GSE6068 (5)	Joseph-Strauss_2007_GSE7393 (6)	Friedlander_2006_GSE3814 (7)	Chu_1998 (8)	Sudarsanam_2000 (9)	Wyrick_1999 (10)	Kuranda_2006_GSE4049 (11)	Levy_2007_GSE6302 (12)	Friedlander_2006_GSE3820 (7)	Munding_2010_GSE24675 (13)	Haugen_2004 (14)	Total
DNA repair (GO:0006281)	Y	Y	Y	Y	Y	Y	Y	Y	Y	Y	Y	Y	Y	Y	Y	15
DNA metabolic process (GO:0006259)	Y	Y	Y	Y	Y	Y	Y	Y	Y	Y	Y	Y	Y	Y	Y	15
cellular protein modification process (GO:0006464)	Y	Y	Y	Y	Y	Y	Y	Y	Y	Y	Y	Y	Y	Y	Y	15
ubiquitin-dependent protein catabolic process (GO:0006511)	Y	Y	Y	Y	Y	Y	Y	Y	Y	Y	Y	Y	Y	Y	Y	15
proteasome-mediated ubiquitin-dependent protein catabolic process (GO:0043161)	Y	Y	Y	Y	Y	Y	Y	Y	Y	Y	Y	Y	Y	Y	Y	15
general amino acid response (GO:0034198)	Y	Y	Y	N	Y	N	N	Y	Y	Y	Y	Y	Y	Y	Y	12
TOR pathway (GO:0031929)	Y	Y	Y	Y	Y	N	N	Y	N	Y	Y	Y	Y	Y	N	11

References:

1. Oromendia AB, Dodgson SE, Amon A. Aneuploidy causes proteotoxic stress in yeast. Genes Dev. 2012;26(24):2696-708. Epub 20121207. doi: 10.1101/gad.207407.112. PubMed PMID: 23222101; PubMed Central PMCID: PMC3533075.

2. Hardwick JS, Kuruvilla FG, Tong JK, Shamji AF, Schreiber SL. Rapamycin-modulated transcription defines the subset of nutrient-sensitive signaling pathways directly controlled by the Tor proteins. Proc Natl Acad Sci U S A. 1999;96(26):14866-70. doi: 10.1073/pnas.96.26.14866. PubMed PMID: 10611304; PubMed Central PMCID: PMC24739.

3. Cheung V, Chua G, Batada NN, Landry CR, Michnick SW, Hughes TR, et al. Chromatin- and transcription-related factors repress transcription from within coding regions throughout the Saccharomyces cerevisiae genome. PLoS Biol. 2008;6(11):e277. doi: 10.1371/journal.pbio.0060277. PubMed PMID: 18998772; PubMed Central PMCID: PMC2581627.

4. Roy S, Wapinski I, Pfiffner J, French C, Socha A, Konieczka J, et al. Arboretum: reconstruction and analysis of the evolutionary history of condition-specific transcriptional modules. Genome Res. 2013;23(6):1039-50. Epub 20130502. doi: 10.1101/gr.146233.112. PubMed PMID: 23640720; PubMed Central PMCID: PMC3668358.

5. Thorsen M, Lagniel G, Kristiansson E, Junot C, Nerman O, Labarre J, et al. Quantitative transcriptome, proteome, and sulfur metabolite profiling of the Saccharomyces cerevisiae response to arsenite. Physiol Genomics. 2007;30(1):35-43. Epub 20070227. doi: 10.1152/physiolgenomics.00236.2006. PubMed PMID: 17327492.

6. Joseph-Strauss D, Zenvirth D, Simchen G, Barkai N. Spore germination in Saccharomyces cerevisiae: global gene expression patterns and cell cycle landmarks. Genome Biol. 2007;8(11):R241. doi: 10.1186/gb-2007-8-11-r241. PubMed PMID: 17999778; PubMed Central PMCID: PMC2258198.

7. Friedlander G, Joseph-Strauss D, Carmi M, Zenvirth D, Simchen G, Barkai N. Modulation of the transcription regulatory program in yeast cells committed to sporulation. Genome Biol. 2006;7(3):R20. Epub 20060308. doi: 10.1186/gb-2006-7-3-r20. PubMed PMID: 16542486; PubMed Central PMCID: PMC1557749.

8. Chu S, DeRisi J, Eisen M, Mulholland J, Botstein D, Brown PO, et al. The transcriptional program of sporulation in budding yeast. Science. 1998;282(5389):699-705. doi: 10.1126/science.282.5389.699. PubMed PMID: 9784122.

9. Sudarsanam P, Iyer VR, Brown PO, Winston F. Whole-genome expression analysis of snf/swi mutants of Saccharomyces cerevisiae. Proc Natl Acad Sci U S A. 2000;97(7):3364-9. doi: 10.1073/pnas.97.7.3364. PubMed PMID: 10725359; PubMed Central PMCID: PMC16245.

10. Wyrick JJ, Holstege FC, Jennings EG, Causton HC, Shore D, Grunstein M, et al. Chromosomal landscape of nucleosome-dependent gene expression and silencing in yeast. Nature. 1999;402(6760):418-21. doi: 10.1038/46567. PubMed PMID: 10586882.

11. Kuranda K, Leberre V, Sokol S, Palamarczyk G, François J. Investigating the caffeine effects in the yeast Saccharomyces cerevisiae brings new insights into the connection between TOR, PKC and Ras/cAMP signalling pathways. Mol Microbiol. 2006;61(5):1147-66. doi: 10.1111/j.1365-2958.2006.05300.x. PubMed PMID: 16925551.
12. Levy S, Ihmels J, Carmi M, Weinberger A, Friedlander G, Barkai N. Strategy of transcription regulation in the budding yeast. PLoS One. 2007;2(2):e250. Epub 20070228. doi: 10.1371/journal.pone.0000250. PubMed PMID: 17327914; PubMed Central PMCID: PMC1803021.
13. Munding EM, Igel AH, Shiue L, Dorighi KM, Treviño LR, Ares M. Integration of a splicing regulatory network within the meiotic gene expression program of Saccharomyces cerevisiae. Genes Dev. 2010;24(23):2693-704. doi: 10.1101/gad.1977410. PubMed PMID: 21123654; PubMed Central PMCID: PMC2994042.
14. Haugen AC, Kelley R, Collins JB, Tucker CJ, Deng C, Afshari CA, et al. Integrating phenotypic and expression profiles to map arsenic-response networks. Genome Biol. 2004;5(12):R95. Epub 20041129. doi: 10.1186/gb-2004-5-12-r95. PubMed PMID: 15575969; PubMed Central PMCID: PMC545798.
